# Vitamin D inhibits bone loss in mice with thyrotoxicosis by activating the OPG/RANKL and Wnt/β-catenin signaling pathways

**DOI:** 10.3389/fendo.2022.1066089

**Published:** 2022-11-30

**Authors:** Dan Xu, Hong-Jiao Gao, Chun-Yan Lu, Hao-Ming Tian, Xi-Jie Yu

**Affiliations:** ^1^ Division of Endocrinology and Metabolism Internal Medicine, West China Hospital, Sichuan University, Chengdu, China; ^2^ Division of Endocrinology & Metabolism, People’s Hospital of Le Shan, Le Shan, China; ^3^ Department of Endocrinology, the Third Affiliated Hospital of Zunyi Medical University (The First People’s Hospital of Zunyi), Zunyi, Guizhou, China

**Keywords:** vitamin D, thyrotoxicosis, bone, OPG/RANKL, Wnt/β-catenin

## Abstract

**Objective:**

Vitamin D and thyroid hormones have crucial roles in bone metabolism. This study aims to explore the effects of vitamin D on bone metabolism in mice with thyrotoxicosis and its mechanisms.

**Methods:**

12-week-old mice were randomly divided into 6 groups (6 mice/group), the control (CON) group, vitamin D (VD) group, low-dose LT4 (Low LT4) group, low-dose LT4+VD (Low LT4+VD) group, high-dose LT4 (High LT4) group, high-dose LT4+VD (High LT4+VD) group, LT4 was provided every day and vitamin D3 every other day for 12 weeks. Thyroid function, 25-hydroxy vitamin D, type I collagen carboxy-terminal peptide (CTX), and type I procollagen amino-terminal peptide were determined. In addition, microcomputed tomography, bone histology and histomorphometry, a three-point bending test, and the mRNA expression of osteoprotegerin (OPG), receptor activator of nuclear factor-κB ligand (RANKL) and β-catenin in bone were conducted.

**Results:**

The BMD of lumbar vertebrae and femur decreased and the bone microstructure was destroyed significantly in thyrotoxicosis mice. Addition of vitamin D improved the BMD and bone microstructure only in the low LT4+VD group. Mice with thyrotoxicosis had a significantly higher level of CTX (*P*<0.05), which was decreased by treatment with vitamin D (*P*<0.05). The eroded surface per bone surface (Er. S/BS) of the cancellous bone and elongated surface/endocortical perimeter (Er. S/E Pm) of the cortical bone significantly increased in the Low LT4 and High LT4 groups (*P*<0.05). Treatment with vitamin D significantly decreased the Er. S/BS and Er. S/E Pm. But, treatment with vitamin D did not significantly improve the toughness and rigidity of bones. The ratio of OPG to RANKL and mRNA expression of β-catenin in the Low LT4+VD group were higher than that in the Low LT4 group (*P*<0.05).

**Conclusion:**

In mice with thyrotoxicosis, treatment with vitamin D can inhibit bone resorption and improve the BMD and trabecular bone architecture by increasing the ratio of OPG to RANKL and upregulating the expression of Wnt/β-catenin.

## Introduction

Thyrotoxicosis is a group of clinical syndromes involving excessive thyroid hormones in blood circulation due to various factors. Excessive thyroid hormones have different effects on bone architecture and bone remodeling (1). Thyrotoxicosis correlate with increased bone turnover markers (BTMs) and decreased bone mineral density (BMD), which increase susceptibility to osteoporotic fractures (2).

OPG/RANKL and Wnt/β-catenin pathways play important roles in bone metabolism (3, 4). The expression OPG and RANKL shows a dynamic balance under normal physiological conditions. Diseases may disrupt the balance between OPG and RANKL, which leads to metabolic abnormalities in bone cell differentiation, maturation, and bone resorption (5). β-catenin is a key molecule in the classical Wnt/β-catenin signaling pathway, which is involved in the maturation, differentiation and apoptosis of osteoblasts. In osteoporosis, the OPG/RANKL and Wnt/β-catenin signalling pathways are inhibited, β-catenin decreases, and the normal ratio of OPG to RANKL is destroyed (6–10). In thyrotoxicosis, the OPG/RANKL system is significantly enhanced, the normal ratio of OPG/RANKL is broken, and bone resorption exceeds bone formation, which is one of the important mechanisms of thyrotoxicosis affecting bone metabolism.

Vitamin D can bidirectional regulate bone formation, absorption and metabolism by binding to VDR receptor through OPG/RANKL, Wnt/β-catenin and other pathways, and maintain the normal growth and development of bone. Its mechanism is complex. It has been found that 1,25(OH)_2_D_3_ promotes the process of bone remodeling by promoting the expression of OPG and RANKL and leading to the change of OPG/RANKL ratio. 1,25 (OH) _2_D_3_ exerts different effects on Wnt/β-catenin pathway in different disease states, and has different effects on bone metabolism.

Thyrotoxicosis and vitamin D are closely related to bone metabolism. However, the influence of vitamin D on bone metabolism and the relevant mechanism in thyrotoxicosis remain unclear. Therefore, this study aims to explore the influence of vitamin D on bone metabolism in mice with thyrotoxicosis and to determine whether it can play a role *via* the OPG/RANKL and Wnt/β-catenin signalling pathways.

## Materials and methods

### Animals

This research protocol was approved by the Laboratory Animal Ethics Committee of West China School of Medicine and West China Hospital, Sichuan University. In the experiment, a total of 36 12-week-old male C57BL/6 mice were purchased from Chengdu Dossy Experimental Animals Co., Ltd. (China). These experimental animals were raised according to the national unified standard (*Laboratory Animals - Nutrients for Formula Feeds* [GB14924.3-2010]; *Laboratory Animals -Hygienic standard for Formula Feeds* [GB/T14924.2-2001]). These mice were randomly divided into 6 groups (6 mice/group): the CON group, the VD group (10 IU/g vitamin D3, every other day), the Low LT4 group (30 µg/100 g LT4, every day), the Low LT4+VD group (30 µg/100 g LT4, every day + 10 IU/g vitamin D3, every other day), the High LT4 group (60 µg/100 g LT4, every day), and the High LT4+VD group (60 µg/100 g LT4, every day + 10 IU/g vitamin D3, every other day). These mice were intragastrically administered LT4 every day and vitamin D3 every other day for 12 continuous weeks. All mice had freely available food and water. The intake of diet and water in the LT4 group was significantly higher than that in the CON and VD groups, and the mice in the high LT4 group had the highest intake. After 12 weeks, these mice were anaesthetized with chloral hydrate. Then, blood samples from these mice were collected from their eyeballs. Next, the mice were euthanized by cervical dislocation to collect their bones. All subsequent analyses were performed by technicians in a blinded manner.

### Serological test

The concentrations of triiodothyronine (T3), thyroxine (T4), TSH, type I collagen carboxy-terminal peptide (CTX), and type I procollagen amino-terminal peptide (PINP) in the mouse serum were determined by enzyme-linked immunosorbent assays (ELISAs) (the ELISA kit was purchased from ZCIBIO Technology Co., Ltd.).

### Microcomputed tomography

Micro-CT was adopted to analyse the distal femur, the fourth lumbar vertebrae, the trabecular bone at the metaphysis far from the growth plate, and the cortical bone at the metaphysis between the femoral head and the distal condyle. In addition, the trabecular bone of the fourth lumbar vertebrae was evaluated around the centre. Additionally, the bone volume/total volume (BV/TV), trabecular number (Tb. N), trabecular separation (Tb. Sp), trabecular thickness (Tb. Th), structural model index (SMI), BMD and other parameters were evaluated.

### Bone histology and histomorphometry

In the experiment, 35 mg·kg^-1^ tetracycline hydrochloride was injected subcutaneously on the 11th and 10th days before euthanasia, and 7.5 mg·kg^-1^ calcein was injected subcutaneously on the 4th and 3rd days before euthanasia to perform fluorescent labelling. Next, the right tibia was embedded and stained without decalcification. Then, the embedded specimens were cut into two kinds of bone slices with thicknesses of 4–5 μm and 9–10 μm by a microtome. Subsequently, toluidine blue staining was performed on thin slices for routine light microscopy to observe the bone architecture and bone tissue cells (osteoblasts and osteoclasts). Thick slices were used to observe and measure fluorescent markers under a fluorescence microscope. The image measurement and analysis system was used for relevant metrological analysis, and some representative photos were obtained.

### Tartrate-resistant acid phosphatase staining

TRAP staining was performed on paraffin sagittal sections of the fourth lumbar vertebrae and left distal femur. The sections were fixed with paraformaldehyde buffered with 4% PBS, decalcified with Osteosoft (Merck, Darmstadt, Germany) for 7 days, and then dehydrated with an ethanol series. The osteoclasts were quantitatively analysed, and some representative photographs were obtained.

### Biomechanical test

The three-point bending test was carried out by the 3-point bending mould of the moving-magnet biomaterial tester (TA Electroforce 3230 III, USA) with a fulcrum span of 8 mm. The midpoint of the femur was selected as the pressure loading point, with a loading rate of 2.0 mm/min. The maximum load, fracture load, elastic load, and corresponding displacement parameters were automatically recorded.

### RNA isolation, reverse transcription, and real-time fluorescence-based quantitative PCR

Total RNA was extracted from the left tibia tissue of mice to perform RNA reverse transcription. The cycle threshold (Ct) of the samples detected during PCR was analysed by Thermo Scientific PikoReal (Thermo). The relative mRNA expression level was calculated by the 2^-△△CT^ method.

### Statistical analysis

The Shapiro−Wilk test was used for the normality test. The measurement data with a normal distribution are expressed as $\bar{x}$ ± s and were analysed by the independent sample t test. The measurement data with a non-normal distribution are expressed as the median (P25-P75) and were analysed by the rank sum test. Histogram analysis and statistical analysis were performed by GraphPad Prism 8 (GraphPad Software, USA). *P*<0.05 indicated a statistically significant difference.

## Results

The serum T4, T3, and TSH concentrations in mice in each group were measured at the end of the study. The Low LT4, High LT4, Low LT4+VD, and High LT4+VD groups had significantly higher concentrations of T3 and T4 but lower concentrations of TSH in the mouse serum than the CON group (both *P*<0.01). Compared with those of the Low LT4 group, the concentrations of T3 and T4 in the High LT4 and High LT4+VD groups increased significantly (*P*<0.01), but the concentration of TSH decreased (*P*<0.01) in the two groups. The concentration of T4 in the VD group was lower than that in the CON group (*P*<0.05), but there was no significant difference in the concentrations of T3 and TSH between these groups (**Table 1**).

**Table 1:** The serum T4, T3, and TSH concentrations in mice in each group.

Group	T3 (ng/ml)	T4 (ng/ml)	TSH (mU/L)
CON	1.507 ± 0.061	43.432 ± 1.705	14.535 ± 1.531
VD	1.486 ± 0.059	40.906 ± 0.971^*^	14.470 ± 1.334
Low LT4	2.910 ± 0.081^**^	51.482 ± 1.755^**^	8.447 ± 1.269^**^
Low LT4+VD	2.866 ± 0.081^**^	50.359 ± 1.850^**^	8.110 ± 0.657^**^
High LT4	3.898 ± 0.070^**^	60.799 ± 1.939^**##^	6.602 ± 0.532^**##^
High LT4+VD	3.861 ± 0.056^**^	59.252 ± 2.901^**##^	6.390 ± 0.359^**^

CON, mice treated with saline; VD, mice treated with vitamin D; Low LT4, mice treated with LT4 (30 µg/100 g. d); Low LT4+VD, mice treated with LT4 (30 µg/100 g. d) and vitamin D (10 IU/g. qod); High LT4, mice treated with LT4 (60 µg/100 g. d); High LT4+VD, mice treated with LT4 (60 µg/100 g. d) and vitamin D (10 IU/g. qod). Data are shown as the means ± SEMs. Compared with the CON group, *P < 0.05, **P < 0.01. Compared with the Low LT4 group, ^##^P < 0.01.

### Bone mineral density is reduced in mice with thyrotoxicosis and restored through vitamin D treatment

**Figure 1:**
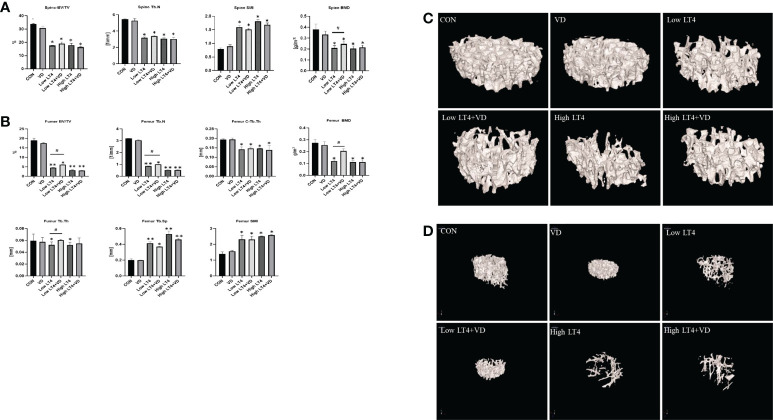
Bone loss and inhibition *via* treatment with vitamin D in mice with thyrotoxicosis. The fourth vertebra and the femur in the CON, VD, Low LT4, Low LT4+VD, High LT4 and High LT4+VD groups were determined by micro-CT. **(A)** Trabecular BV/TV, Tb. N, SMI and BMD of the spine. **(B)** BV/TV, Tb. N, C-Tb. Th, BMD, Tb. Th, Tb. Sp and SMI of the femur. **(C)** Representative 3D reconstructions of the trabeculae. **(D)** Representative 3D reconstructions of the femur. **(A, B)** Data are shown as the means ± SEMs (n=6). Compared with the CON group. *P < 0.05, **P < 0.01. Compared with the Low LT4 group, ^#^ *P* < 0.05.

The lumbar vertebrae micro-CT results (**Figure 1A**) showed that the BV/TV, Tb. N and BMD in Low LT4, Low LT4+VD, High LT4, and High LT4+VD groups significantly decreased compared with those in CON group (*P*<0.05). However, the BMD in the Low LT4+VD group increased by 17.3% compared with that in the Low LT4 group (*P*<0.05). The SMI in the Low LT4, Low LT4+VD, High LT4, and High LT4+VD groups increased by 1.0, 0.91, 1.3, and 1.1 times, respectively, compared with that in the CON group (*P*<0.05).

The femur micro-CT results (**Figure 1B**) showed that the BV/TV, Tb. N, C-Tb. Th and BMD in the Low LT4, Low LT4+VD, High LT4, and High LT4+VD groups significantly decreased compared with those in CON group (*P*<0.05). The BV/TV, Tb. N, BMD in the Low LT4+VD group increased by 36.29%, 17.22% and 100%, respectively, compared with those in the Low LT4 group (*P*<0.05). The Tb. Th in the Low LT4 and High LT4 groups decreased by 26.68% and 24.15%, respectively, compared with that in the CON group (*P*<0.05). The Tb. Th in the Low LT4+VD group increased by 15.14% compared with that in the Low LT4 group (*P*<0.05). The Tb. Sp and SMI in the Low LT4, Low LT4+VD, High LT4, and High LT4+VD groups significantly increased compared with those in the CON group (*P*<0.05), but there was no significant difference between Low LT4+VD group and Low LT4 group.

The micro-CT images of the lumbar vertebrae and femur (**Figures 1C, D**) showed that the CON and VD groups had a normal trabecular bone architecture, which was closely arranged. Decreased Tb. N, trabecular fracture, disordered arrangement, and sparse bone architecture were observed in the Low LT4, Low LT4+VD, High LT4, and High LT4+VD groups, especially in the High LT4 group. The Low LT4+VD group and the High LT4+VD group had an improved bone architecture compared with the Low LT4 group and the High LT4 group, respectively.

Our research suggested that vitamin D can improve the BMD in a state of mild thyrotoxicosis state, but the protective effect was not obvious in a state of severe thyrotoxicosis.

### Bone resorption is increased in mice with thyrotoxicosis and inhibited through treatment with vitamin D

**Figure 2:**
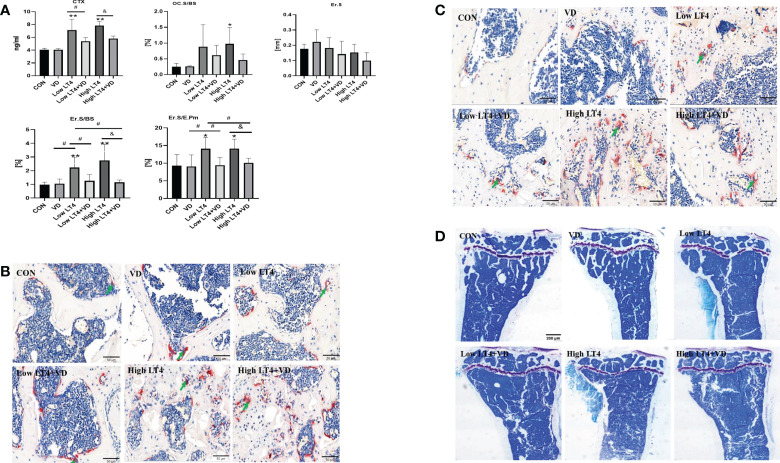
Bone resorption is increased in mice with thyrotoxicosis and suppressed through treatment with vitamin D. **(A)** Bone resorption parameters were determined in the CON, VD, Low LT4, Low LT4+VD, High LT4 and High LT4+VD groups. CTX, type I collagen carboxy-terminal peptide; Oc. S/BS, osteoclast surface per bone surface; Er. S, eroded surface; Er. S/BS, eroded surface per bone surface; Er. S/E. Pm, elongated surface/endocortical perimeter. **(B)** Representative TRAP staining on vertebral slides in the CON, VD, Low LT4, Low LT4+VD, High LT4 and High LT4+VD groups. **(C)** Representative TRAP staining on femur slides in the CON, VD, Low LT4, Low LT4+VD, High LT4 and High LT4+VD groups. **(D)** Toluidine blue staining of cancellous bone in the metaphysis of the upper tibia of mice in the CON, VD, Low LT4, Low LT4+VD, High LT4 and High LT4+VD groups. **(A)** Data are shown as the means ± SEMs (n=6). Compared with the CON group, *P < 0.05, **P < 0.01. Compared with the Low LT4 group, ^#^ *P* < 0.05, compared with the High LT4 group, ^&^ *P* < 0.05.

The concentration of CTX in the Low LT4 and High LT4 groups was significantly higher than that in the CON group (*P*<0.01). The concentration of CTX in the Low LT4+VD group was significantly lower than that in the Low LT4 group (*P*<0.05), and that in the High LT4+VD group was significantly lower than that in the High LT4 group (*P*<0.05) (**Figure 2A**). Bone resorption was evaluated by bone histomorphometry (**Figure 2A**), and the results showed that the osteoclast surface per bone surface (Oc. S/BS) and eroded surface (Er. S) of the cancellous bone in the upper tibia of mice in the VD, Low LT4, Low LT4+VD, and High LT4+VD groups were not significantly different from those in the CON group. The Oc. S/BS in the High LT4 group increased by 2.93 times compared with that in the CON group (*P*<0.05). The Er. S/BS and Er. S/E. Pm of the cortical bone in the middle tibia in the Low LT4 and High LT4 groups increased compared with those of the CON group (*P*<0.05); Vitamin D supplementation decreases the Er. S/BS and Er. S/E. Pm (*P*<0.05). The TRAP staining results showed that larger and more osteoclasts were detected in the lumbar vertebrae and distal femur of mice in the Low LT4, Low LT4+VD, High LT4, and High LT4+VD groups, especially in the High LT4 group (**Figures 2B, C**).

The toluidine blue staining results of bone tissues of mice in each group (**Figure 2D**) showed that the trabecular bone architecture in the CON and VD groups was clear and compact, with many uniform trabecular bones and favorable continuity. The Tb. N in the Low LT4 group decreased significantly, and the trabecular bone architecture became sparse, with fracture and discontinuity in the trabecular bone. There was almost no integrated trabecular bone architecture in the High LT4 group, with a large area without the trabecular bone. The Tb. N in the Low LT4+VD group increased compared with that in the Low LT4 group, with a higher continuity. Although both the High LT4+VD and High LT4 groups had more bone defects, there were few incomplete trabecular bones in the High LT4+VD group.

Thus, our study showed that LT4-induced thyrotoxicosis enhanced bone resorption in proportion to the severity of thyrotoxicosis, and vitamin D supplementation inhibited bone resorption.

### Thytoxicosis-induced bone formation is not obviously changed by vitamin D treatment

**Figure 3:**
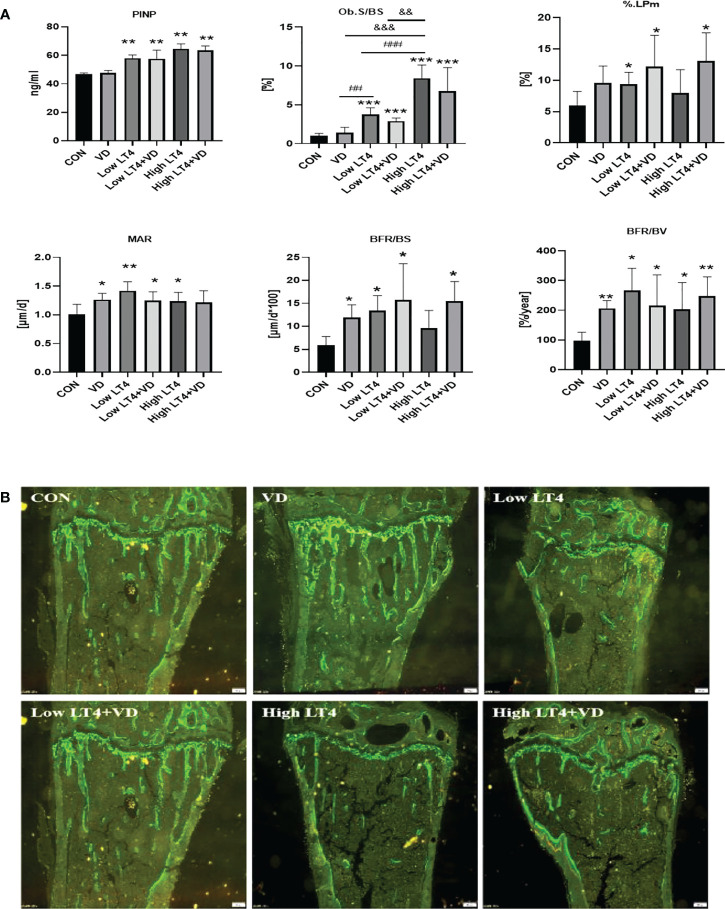
Thytoxicosis-induced bone formation is not obviously changed by vitamin D treatment. **(A)** Bone formation parameters were determined in the CON, VD, Low LT4, Low LT4+VD, High LT4 and High LT4+VD groups. P1NP, procollagen type 1 N-terminal propeptide; Ob. S/BS, osteoblast surface per bone surface; %L. Pm, % label perimeter. MAR, mineral apposition rate of cancellous. BFR/BS, bone formation rate/bone surface. BFR/BV, bone formation rate/bone volume. **(B)** Representative calcein double labelling of the upper tibia in mice. **(A)** Data are shown as the means ± SEMs (n=6). Compared with the control group, *P<0.05, **P<0.01, ***P<0.001. Compared with the Low LT4 group, ^#^ *P* < 0.05, ^##^ *P* < 0.01,001, ^###^P < 0.001. compared with the High LT4 group, ^&^ *P* < 0.05, ^&&^ *P* < 0.01, ^&&&^P < 0.001.

The P1NP in the Low LT4, Low LT4+VD, High LT4, and High LT4+VD groups increased significantly compared with that in the CON group (*P*<0.01). Vitamin D supplementation had no significant effect on serum P1NP level in thyrotoxicosis mice (**Figure 3A**). Bone formation was evaluated by bone histomorphometry (**Figure 3A**), and the results showed that the Ob. S/BS of the cancellous bone in the upper tibia of mice in the Low LT4, Low LT4+VD, High LT4, and High LT4+VD groups increased by 2.79, 1.93, 7.46, and 5.80 times, respectively, compared with that of the CON group (*P*<0.001). The % label perimeter (% L. Pm) in the Low LT4, Low LT4+VD and High LT4+VD groups increased by 56.46%, 103% and 118%, respectively, compared with that of the CON group (*P*<0.05). The mineral apposition rate (MAR), bone formation rate/bone surface (BFR/BS) and bone formation rate/bone volume (BFR/BV) in the VD, Low LT4, Low LT4+VD, and High LT4 groups increased compared with that of the CON group (*P*<0.05). There was no significant difference in the Ob. S/BS, %L. Pm, MAR, and BFR/BV between the Low LT4+VD group and the Low LT4 group and between the high LT4+VD group and the High LT4 group. There was no significant difference in the mineralized surface/bone surface (MS/BS) of the cortical bone of the middle tibia, endocortical MS/BS, MAR, and BFR/BS of mice and in all groups. The periosteal MAR and BFR/BS could not be detected in the Low LT4, Low LT4+VD, High LT4, and High LT4+VD groups.

The bone histomorphometric fluorescence analysis of the upper tibia of mice in each group (**Figure 3B**) showed that there was a clear trabecular bone architecture, normal Tb. N, and increasingly brighter fluorescence in the CON group; the interlabel width was faintly visible. The VD group had a normal Tb. N and increasingly brighter fluorescence. The interlabel width was clearly visible, and it was larger than that in the CON group. In the LT4 group, a significantly decreased Tb. N and an enlarged medullary cavity, with dim fluorescence, were observed. The interlabel width was visible. In the LT4+VD group, the Tb. N, which was slightly higher than that in the LT4 group, with dim fluorescence, was observed. The interlabel width was similar to that in the LT4 group. In the High LT4 group, there was a further decreased Tb. N and a large medullary cavity, with reduced fluorescence. The interlabel width was visible and larger than that of the CON group. There was a slightly increased Tb. N in the High LT4+VD group compared with the High LT4 group, with increased fluorescence. The interlabel width was clearly visible.

Our study showed that bone formation enhanced in thyrotoxicosis mice, and vitamin D supplementation had no significant effect on bone formation.

### Bone strength is reduced in mice with thyrotoxicosis and not restored through vitamin D treatment

According to the three-point bending test results, the elastic load of mice in the Low LT4, Low LT4+VD, High LT4, and High LT4+VD groups decreased by 34.73%, 45.62%, 41.11%, and 35.89%, respectively, compared with that in the CON group (*P*<0.01). The elastic deflection of mice in the Low LT4, Low LT4+VD, High LT4, and High LT4+VD groups was not significantly different from that in the CON groups. The fracture load of mice in the Low LT4, High LT4, and High LT4+VD groups decreased by 21.26%, 44.31%, and 58.12%, respectively, compared with that in the CON group (*P*<0.01). The maximum deflection of mice in the High LT4 and High LT4+VD groups decreased by 28.19% and 27.98%, respectively, compared with that in the CON group (*P*<0.05). The maximum load of mice in the Low LT4, High LT4, and High LT4+VD groups decreased by 18.34%, 31.39%, and 27.44%, respectively, compared with that in the CON group (*P*<0.01). There was no significant difference in the elastic load, elastic deflection, fracture load, maximum load, or maximum deflection between the Low LT4+VD group and the Low LT4 group or between the High LT4+VD group and the High LT4 group (**Table 2**).

**Table 2:** Comparison of biomechanical indexes of mice in each group.

Group	Elastic load (N)	Elastic deflection (mm)	Fracture load (N)	Maximum deflection (mm)	Maximum load (N)
CON	15.806 ± 2.251	0.648 ± 0.062	17.122 ± 1.917	1.444 ± 0.307	20.344 ± 1.075
VD	14.423 ± 3.563	0.614 ± 0.052	23.205 ± 6.650	1.082 ± 0.353	24.860 ± 5.287
Low LT4	10.316 ± 0.959^**^	0.553 ± 0.107	13.482 ± 0.850^**^	1.067 ± 0.269	16.612 ± 2.093^**^
Low LT4+VD	8.595 ± 1.514^**^	0.658 ± 0.134	14.932 ± 3.286	1.083 ± 0.436	17.553 ± 3.491
High LT4	9.307 ± 1.661^**^	0.606 ± 0.059	9.535 ± 3.506^**^	1.037 ± 0.260^*^	13.958 ± 2.602^**^
High LT4+VD	10.134 ± 1.630^**^	0.541 ± 0.712^*^	7.170 ± 1.778^**^	1.040 ± 0.194^*^	14.762 ± 2.803^**^

Biomechanical parameters in the CON, VD, Low LT4, Low LT4+VD, High LT4 and High LT4+VD groups. Data are expressed as the means ± SEMs (n = 5). Statistical significance was calculated using the Mann–Whitney U test for nonparametric variables and Student’s t test. Compared with the CON group, *P < 0.05, **P < 0.01.

### Vitamin D protects against thyrotoxicosis-induced bone loss in mice through the OPG/RANKL and Wnt/β-catenin pathways

**Figure 4:**
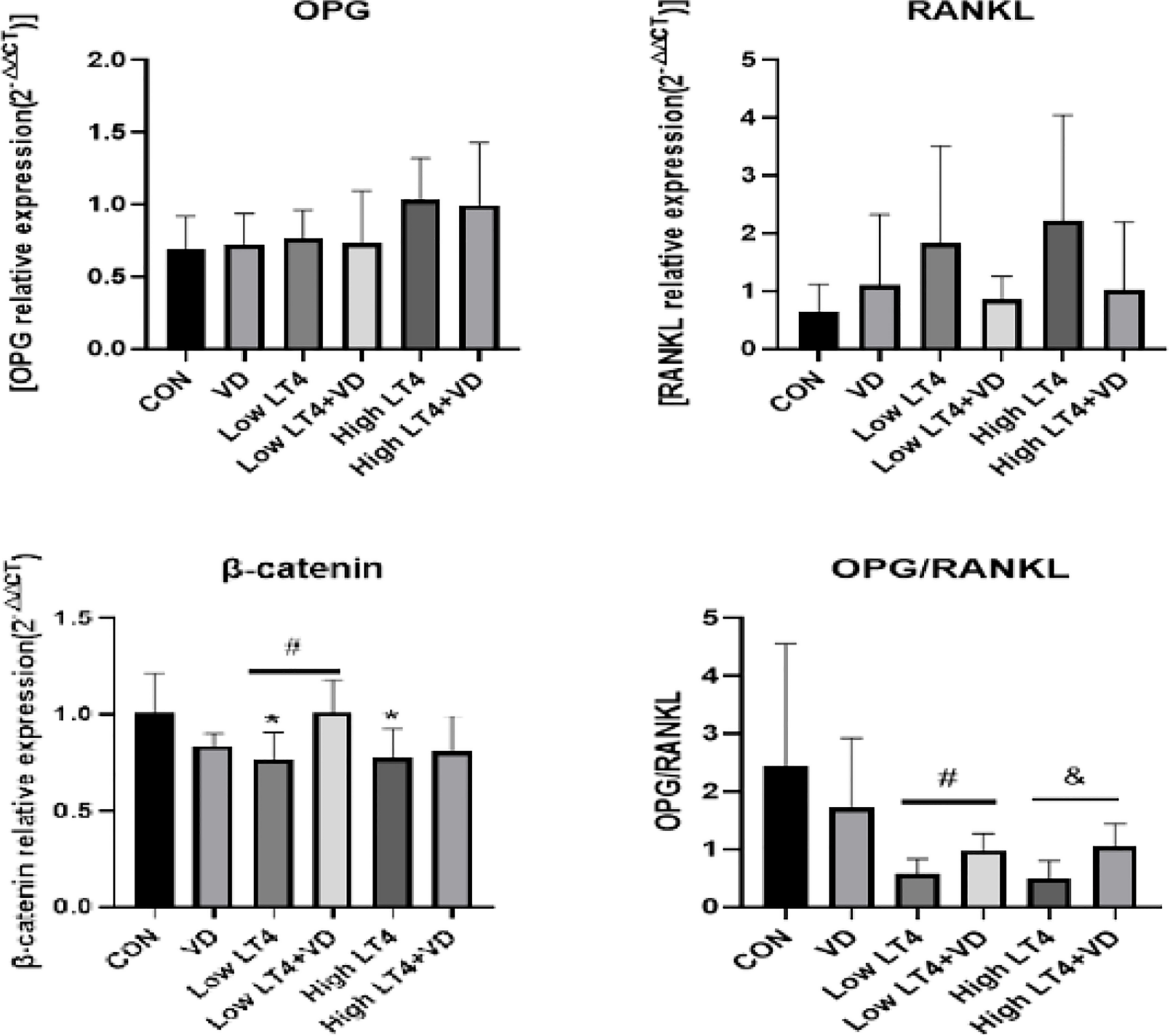
Analysis of the OPG/RANKL and Wnt/β-catenin pathways. Quantitative RT−PCR analysis of OPG, RANKL and β-catenin. The results were calculated based on the ΔΔCT method and normalized to the β-actin mRNA levels. OPG/RANKL ratio compared with that of the CON group, *P < 0.05,. compared with the Low LT4 group, ^#^ *P* < 0.05. Compared with the High LT4 group, ^&^ *P* < 0.05.

In this study, the mRNA expression of OPG, RANKL, and β-catenin in mouse bones was detected by real-time PCR. The results (**Figure 4**) showed that there were no significant difference in the expression of OPG and RANKL among all groups. The ratio of OPG to RANKL was further compared among these groups. The ratio of OPG to RANKL in the Low LT4, Low LT4+VD, High LT4, and High LT4+VD groups decreased by 45.10%, 17.04%, 67.70%, and 47.65%, respectively, compared with that in the CON group. The ratio of OPG to RANKL in the Low LT4+VD group was 51.11% higher than that in the Low LT4 group, and the ratio of OPG to RANKL in the High LT4+VD group was 62.10% higher than that in the High LT4 group (*P*<0.05). The mRNA expression of β-catenin in the Low LT4 and High LT4 groups decreased by 24.8% and 24.01%, respectively, compared with that in the CON group (*P*<0.05). The mRNA expression of β-catenin in the Low LT4+VD group was 32.46% higher than that in the Low LT4 group (*P*<0.05). However, the mRNA expression of β-catenin in the High LT4+VD group was only slightly higher than that in the High LT4 group, without a significant difference.

## Discussion

Thyroid hormones is an important factor to bone homeostasis (11–13). Excessive T3 can inhibit the replication of osteoblasts, increase the number and activity of osteoclasts, and accelerate bone metabolism. These effects can make bone resorption faster than bone formation and decrease BMD and bone mineralization, thus inducing osteoporosis. Vitamin D is an important steroid hormone involved in the calcium and phosphorus metabolism of bone minerals, and its level is closely related to BMD. Adequate vitamin D helps maintain a positive calcium balance, which reduces bone turnover imbalance and bone loss. In a randomized, placebo-controlled study, researchers found that supplementing vitamin D can decrease the bone turnover level, reduce the bone loss rate, and increase the BMD of the lumbar vertebrae and hip (14, 15).

To date, there are few studies on the correlation between vitamin D and bone metabolism in thyrotoxicosis. In this study, an LT4-induced thyrotoxicosis mouse model was successfully established. The results confirmed that the CTX and P1NP increased significantly in the mice with thyrotoxicosis. Supplementation with vitamin D could significantly decrease CTX and slightly decrease P1NP. Yoshizawa et al. (16) also found that bone formation and bone mineralization in mice with the knockout of vitamin D receptors were seriously affected. With the gradual decrease in the vitamin D level, the levels of PINP and CTX were improved, and bone metabolism was enhanced. In addition, PINP and CTX were negatively correlated with 25OHD, which indicated that the inhibition of bone resorption was reduced with the decrease in the vitamin D level (17). In this study, the osteoclast-specific TRAP staining results revealed that the number of osteoclasts in the trabecular bone of the lumbar vertebrae and distal femur of the mice with thyrotoxicosis increased significantly. This finding indicated that bone resorption in the mice with thyrotoxicosis increased significantly, and supplementation with vitamin D reduced the number of osteoclasts in these mice. In addition, the micro-CT results showed that the BMD of the lumbar vertebrae and femur in the mice with thyrotoxicosis decreased and the bone architecture was destroyed, which was similar to the findings of most studies (18, 19). Treatment with vitamin D significantly improved the BMD and trabecular bone architecture of the lumbar vertebrae and femur in the mice with low LT4-induced thyrotoxicosis. However, treatment with vitamin D had no significant protective effect on bone loss of the mice with thyrotoxicosis in the High LT4 group.

Moreover, the influences of vitamin D on bone histomorphometry in the mice with thyrotoxicosis were observed. An analysis of cancellous bone showed that Ob. S/BS, Er. S/BS, and Oc. S/BS increased significantly in these mice. This result indicated that the bone formation rate and bone resorption increased in the mice with thyrotoxicosis. Supplementation with vitamin D mainly inhibited bone resorption but had no significant effect on the bone formation rate in these mice. The cortical bone in the mice with thyrotoxicosis was also explored. The results demonstrated that the periosteal MAR and BFR could not be detected. However, the endocortical perimeter increased, and bone resorption increased significantly. Treatment with vitamin D reduced the endocortical perimeter and inhibited bone resorption to a certain extent. We also found that treatment with vitamin D in mice with normal thyroid functions could increase the %L. Pm, the endocortical MAR and BFR of cancellous bone and cortical bone, and the periosteal %L. Pm of cortical bone. However, treatment with vitamin D did not significantly increase the values of these indexes in the mice with thyrotoxicosis. Abu E O et al. (20) proved that the combination of thyroid hormones with nuclear receptors and membrane receptors of osteoblasts could exert biological effects by affecting cell replication and protein synthesis, while high-dose thyroid hormones could inhibit cell replication. Thyroid hormones can enhance the activity of osteoclasts under the action of local mediators. This finding indicated that the more severe the thyrotoxicosis, the higher the level of bone resorption and bone formation is, and the enhancement of bone resorption was greater than that of bone formation, which was similar to the results of this study. Therefore, treatment with vitamin D mainly inhibited bone resorption of cancellous bone and cortical bone in mice with thyrotoxicosis but had no significant influence on bone formation. This finding may be explained by the obvious enhancement of bone resorption in the mice with thyrotoxicosis, which far exceeded the effect of bone formation.

Biomechanical analysis of bones is an important method to measure bone strength by evaluating the mechanical characteristics and biological effects of bones under external forces. It is also a direct and reliable method to evaluate bone quality. In this study, a three-point bending test was conducted to explore the influence of vitamin D on bone rigidity and strength in thyrotoxicosis. The test results revealed that thyrotoxicosis could significantly decrease the elastic load, fracture load, and maximum load of bones of these mice. This result indicated that thyrotoxicosis could seriously affect the toughness and rigidity of bones. Supplementation with vitamin D could enhance the toughness and rigidity of bones, but the improvement was not significant. This results may be due to an insufficient duration of treatment with vitamin D. In addition, bone resorption may be enhanced in persistent thyrotoxicosis. Although vitamin D supplementation can improve bone density and bone microstructure, it cannot significantly enhance bone strength. Drugs inhibiting bone resorption or promoting bone formation may be required to further strengthen bone strength. The influence of thyrotoxicosis on bone strength in mice has been explored in several studies (18, 19). The results showed that thyrotoxicosis decreased bone strength in mice, which was similar to the results of this study. In a study by Williamson L et al. (21), adult mice with vitamin D3 in their diet had a greater ductility and toughness of the tibia and a larger volume fraction of the trabecular bone. Supplementation with vitamin D3 may result in higher peak bone mass. In a study based on a rat model, the rigidity and strength of bones in vitamin D deficient rats decreased, and supplementation with active vitamin D enhanced the response of cortical bones to mechanical load (22, 23).

Currently, the mechanism related to the treatment with vitamin D on bone metabolism in thyrotoxicosis remains unclear. To further identify the possible mechanism of the effect of vitamin D on bone metabolism in mice with thyrotoxicosis, we explored the important factors in the OPG/RANKL and Wnt/β-catenin bone metabolic pathways. In addition, the mRNA expression of these factors was determined to identify whether vitamin D could affect bone metabolism in the mice with thyrotoxicosis *via* the above pathways. The results showed that β-catenin expression decreased in the mice with thyrotoxicosis. Although there was no significant difference in the expression of OPG and RANKL among all groups, there was an upwards trend in the expression of these factors in the mice with thyrotoxicosis, which indicated enhanced bone resorption. Supplementation with vitamin D can increase the expression of β-catenin and significantly change the ratio of OPG to RANKL. This finding suggested that vitamin D supplementation may reduce the bone mass loss caused by thyrotoxicosis *via* the OPG/RANKL and Wnt/β-catenin signaling pathways. Another study (24) confirmed that vitamin D could directly act on osteoblasts and osteoclast precursor cells and affect the differentiation and formation of osteoblasts and osteoclasts by stimulating or inhibiting the expression of genes related to bone metabolism, thus playing a role in regulating bone homeostasis. OPG is a competitive inhibitor of RANKL, and it can inhibit the activity, differentiation, and apoptosis of osteoclasts by blocking the combination of RANKL and RANK, thus inhibiting bone resorption and preventing bone mass loss (25). Several animal studies (26, 27) showed that severe osteoporosis occurs in mice with OPG knockout, and the BMD of these mice increased after the injection of OPG-Fc. The expression of RANKL is regulated by many signaling molecules and hormones, including 1,25(OH)_2_D and Wnt signaling ligand (28–30). Another report showed that osteoblasts can respond to bone formation by expressing RANKL, and 1,25(OH)_2_D plays a crucial role in inducing the expression of RANKL in osteoblasts (31). Takahashi N et al. found that supplementation with active vitamin D could inhibit the expression of RANKL in osteoblasts and increase the BMD of mice (32, 33). Another study showed that supplementation with 1,25(OH)_2_D could inhibit the expression of RANKL in patients with rheumatoid arthritis (34). Therefore, the influence of 1,25(OH)_2_D on RANKL, which may vary in different diseases, remains controversial (35). As shown in previous studies, the ratio of OPG to RANKL can reflect bone formation and bone resorption. Additionally, 1,25(OH)_2_D can promote the generation of OPG and RANKL and cause changes in the ratio of OPG to RANKL, thus promoting bone remodeling. In a study on diabetic rats (36), vitamin D3 was found to reduce vascular endothelial injuries in these rats by regulating the ratio of OPG to RANKL. These findings are similar to the results of this study. β-catenin is involved in the differentiation, maturation, and apoptosis of osteoblasts, and it is a key molecule in the classical Wnt/β-catenin signaling pathway. Although the deletion of β-catenin genes in mature osteoblasts would not have a direct impact on osteoblasts, it would indirectly increase the number and activity of osteoclasts. The destroyed balance between the osteogenic and osteoclastic processes would affect the bone metabolic process (37). Therefore, the OPG/RANKL and Wnt/β-catenin signaling pathways play an important role in the pathogenesis of osteoporosis. In this study, the effects of vitamin D on the OPG/RANKL and Wnt/β-catenin pathways related to bone metabolism were explored in mice with thyrotoxicosis. The results demonstrated that the expression of β-catenin decreased, the expression of OPG and RANKL increased, and the activity of osteoclasts increased significantly. Supplementation with vitamin D increased the expression of β-catenin and changed the ratio of OPG to RANKL. This finding suggested that vitamin D supplementation might reduce the bone mass loss caused by thyrotoxicosis through the OPG/RANKL and Wnt/β-catenin signaling pathways. However, there is no report related to this finding in previous studies.

This study has some limitations. First, treatment with vitamin D may have potential effects on the thyroid state. Second, thyrotoxicosis and vitamin D supplementation lasted for only 12 weeks, which could not completely simulate chronic thyrotoxicosis in the human body. Third, we selected only male mice in the study to avoid the influence of sex hormones, which may also affect the thyroid state. Fourth, the influence of vitamin D on the OPG/RANKL and Wnt/β-catenin pathways was only explored at the RNA level. Thus, it is necessary to conduct further explorations at the protein level.

In conclusion, LT4-induced thyrotoxicosis could significantly increase bone resorption *via* high bone turnover. This process would decrease the BMD of bones, destroy bone microstructure, and reduce the toughness and rigidity of bones. These conditions are directly proportional to the severity of thyrotoxicosis. Treatment with vitamin D can inhibit bone resorption in mice with thyrotoxicosis and significantly improve the BMD and trabecular bone architecture of the lumbar vertebrae and femur, which may be achieved by the OPG/RANKL and Wnt/β-catenin signaling pathways.

## Data availability statement

The original contributions presented in the study are included in the article/supplementary material. Further inquiries can be directed to the corresponding authors.

## Ethics statement

The animal study was reviewed and approved by the Ethics Committee of West China Hospital of Sichuan University (Approval No.: 2016 Annual Review (130).

## Author contributions

DX and H-JG had full access to all of the data in the study and takes responsibility for the integrity of the data and the accuracy of the data analysis. Study concept and design: DX, H-JG, C-YL, and H-MT. Acquisition of data: DX and H-JG. Analysis and interpretation of data: All authors. Drafting of the manuscript: DX. Critical revision of the manuscript for important intellectual content: All authors. Statistical analysis: DX and H-JG. Study supervision: C-YL and H-MT. All authors contributed to the article and approved the submitted version.

## Conflict of interest

The authors declare that the research was conducted in the absence of any commercial or financial relationships that could be construed as a potential conflict of interest.

## Publisher’s note

All claims expressed in this article are solely those of the authors and do not necessarily represent those of their affiliated organizations, or those of the publisher, the editors and the reviewers. Any product that may be evaluated in this article, or claim that may be made by its manufacturer, is not guaranteed or endorsed by the publisher.
